# Automated Safety Plan Scoring in Outpatient Mental Health Settings Using Large Language Models: Exploratory Study

**DOI:** 10.2196/79010

**Published:** 2026-01-08

**Authors:** Hayoung K Donnelly, Gregory K Brown, Kelly L Green, Ugurcan Vurgun, Sy Hwang, Emily Schriver, Michael Steinberg, Megan E Reilly, Haitisha Mehta, Christa Labouliere, Maria A Oquendo, David Mandell, Danielle L Mowery

**Affiliations:** 1 Department of Psychiatry, University of Pennsylvania, Philadelphia, PA, United States; 2 Institute for Biomedical Informatics, University of Pennsylvania, 3700 Hamilton WalkPhiladelphia, PA, 19104, United States, 1-215-746-6677; 3 University of Pennsylvania Health System, Philadelphia, PA, United States; 4 Department of Psychiatry, Columbia University, New York, NY, United States; 5 Department of Biostatistics, Epidemiology and Informatics, University of Pennsylvania, Philadelphia, PA, United States

**Keywords:** suicide, mental health informatics, generative AI, clinician support, patient-reported data, artificial intelligence

## Abstract

**Background:**

The safety planning intervention (SPI) is a suicide prevention intervention that results in a written plan to help patients reduce suicide risk. High-quality safety plans—that is, those that are the most complete, personalized, and specific—are more effective in reducing suicide risk. Measuring SPI quality is labor-intensive, which means that clinicians rarely get specific, actionable feedback on their use of the SPI.

**Objective:**

This study aimed to develop the Safety Plan Fidelity Rater, an automated tool that assesses the quality of written safety plans leveraging 3 large language models (LLMs)—GPT-4, LLaMA 3, and o3-mini.

**Methods:**

Using 266 deidentified safety plans from outpatient mental health settings in New York, LLMs analyzed four key steps: warning signs, internal coping strategies, making the environment safe, and reasons for living. We compared the predictive performance of the three LLMs, optimizing scoring systems, prompts, and parameters.

**Results:**

Findings showed that LLaMA 3 and o3-mini outperformed GPT-4, with different step-specific scoring systems recommended based on weighted *F*_1_-scores.

**Conclusions:**

These findings highlight LLMs’ potential to provide clinicians with timely and accurate feedback on safety plan quality, which could greatly improve its implementation in community practice.

## Introduction

The safety planning intervention (SPI) is a widely used, evidence-based intervention to prevent suicide [1]. It is designed to help patients develop awareness of their personal warning signs and pre-plan specific strategies to use to prevent and manage acute suicidal crises [1]. The written safety plan that results from SPI has six steps: (1) identifying internal warning signs of an impending acute suicidal crisis; (2) identifying distracting activities the individual can do by themselves; (3) identifying people and social settings that can be used for distraction; (4) identifying friends and family that the individual can reach out to for help; (5) identifying professionals and professional resources the individual can reach out to for help; and (6) developing an action plan to reduce access to lethal means. In some cases, the written safety plan includes an optional Step 7, which identifies the patient’s reasons for living. SPI reduces suicidal behavior following emergency department discharge by almost half compared to usual care [2]. Unfortunately, evidence suggests that clinicians do not implement SPI as intended, which likely decreases its effectiveness in reducing suicidal behavior [3-5].

Providing real-time feedback to clinicians could greatly enhance their use of the SPI [4,6,7]; however, the current state of the science requires direct observation by expert clinicians to provide this feedback. Even providing useful, asynchronous feedback based on recorded SPI sessions is challenging. While an alternative approach of rating the quality of the written safety plan is more scalable, using human coders still requires significant time and training [8].

Applying large language models (LLMs) in mental health care services can improve service delivery [9]. Existing LLM applications have primarily focused on risk prediction, rather than on evaluating the quality of clinical care. One study found that only 15% of LLM applications in mental health research focused on supporting treatment and intervention practices, whereas 47% concentrated on risk prediction [10]. Even among the few studies on treatment and intervention support, most have focused on clinical documentation summarization and information extraction, rather than providing feedback on treatment quality. There has been some attempt to use LLMs to improve suicide prevention. For example, one study evaluated the presence of the components of the SPI documented in the electronic health record using traditional rule-based natural language processing methods, finding that computational approaches can be used to understand suicide prevention interventions in real-world settings [11]. This effort could be further advanced by applying LLM techniques to assess the fidelity of written safety plans in a more nuanced and contextually sensitive manner without the need for ontological reasoning.

In this pilot study, we developed and pilot-tested an automated Safety Plan Fidelity Rater (SPFR), an artificial intelligence (AI) aid for scoring safety plans. We developed the SPFR by comparing three LLMs based on their weighted *F*_1_-scores and optimizing their prompts and parameters to maximize performance. We hypothesized that o3-mini would outperform GPT-4 and LLaMA 3 in effectively scoring written safety plans compared to scores assigned by trained clinical coders because of its advanced reasoning capabilities.

## Methods

### Data

We analyzed 266 deidentified written safety plans collected as part of a Zero Suicide implementation project [12]. Safety plans were collected from patients who received the SPI across 61 outpatient mental health clinics in New York State. Patient demographic data were not available in this study; only the written responses on the safety plans were used for analysis. Written responses on the safety plan are intended to be collaboratively developed by the clinician and the patient together. Coders assessed safety plan response quality using the SPI Scoring Algorithm (SPISA; GKB, unpublished data, 2023). The SPISA consists of 20 items measuring the quality and completeness of written responses across seven steps of the safety plan form: warning signs, internal coping strategies, social contacts and social settings, social contacts for a crisis, professionals for a crisis, making the environment safe, and reasons for living*.* The quality of response codes evaluates how specific and personalized the response is, as well as its relevance to each step’s purpose. Completeness of response codes evaluates whether a response is present or absent on a given line. We focused on developing an automated scoring tool for the quality of responses, particularly for the following four steps of the Safety Plan: warning signs, internal coping strategies, making the environment safe, and reasons for living*.* The remaining three steps were excluded due to a high number of missing entries following the deidentification process, as they often contained personal information (eg, names and phone numbers) that were redacted in compliance with the Health Insurance Portability and Accountability Act of 1996 using the Safe Harbor method.

Below are the definitions of the four target steps, along with fictitious but realistic examples:

- Warning signs*:* specific thoughts, feelings, physiological states, or behaviors that are associated with the development of a suicidal crisis (eg, “not sleeping well,” “my heart starts pounding faster”).
- Internal coping strategies*:* activities the patient can engage in by themselves that distract from suicidal urges and allow time for the crisis to pass (eg, “write in my journal,” “listening to the Beatles”).
- Making the environment safe*:* an action plan that the client will complete to reduce access to lethal means (eg, “get rid of unused medication,” “lock up my firearm and give the key to my brother”).
- Reasons for living: things that matter most to clients and give them a sense of purpose and motivation to continue living (eg, “my family,” “I want to go to college, become a nurse, and help people”).

The maximum possible number of responses differed for each step: 3 for warning signs, 3 for internal coping strategies, 2 for making the environment safe, and 1 for reasons for living. If more than the maximum number of responses were present for a given step, the SPISA dictates for the highest coded responses to be chosen up to the maximum number (eg, if for warning signs, 4 responses were given of 3, 3, 2, and 1, then the top 3 highest scores of 3, 3, and 2 are chosen). Among 2210 individual responses across four steps from the 266 safety plan forms, we observed 772 responses for warning signs, 770 responses for internal coping strategies, 405 responses for making the environment safe, and 263 responses for reasons for living*.*

**Figure 1.**
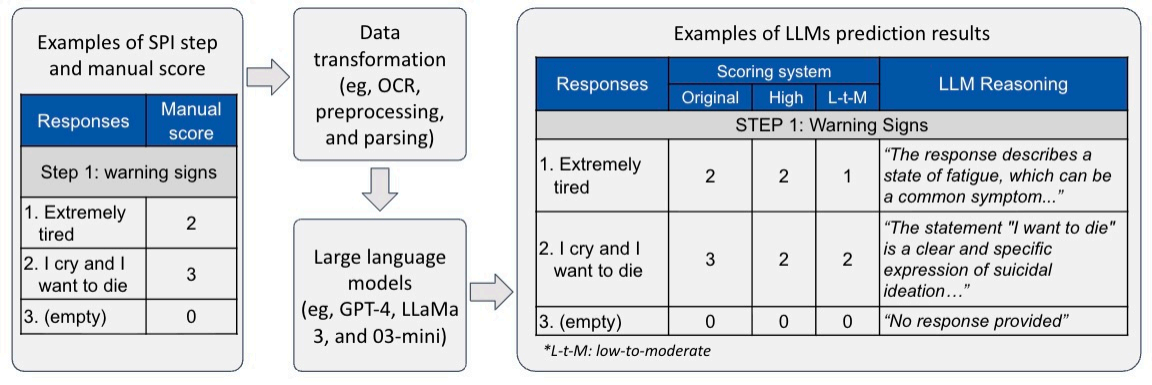
Workflow of the Safety Plan Fidelity Rater development. LLM: large language model; OCR: optical character recognition; SPI: safety planning intervention.

In Figure 1, we present a workflow diagram illustrating an example of the safety plan steps, the SPFR development, and output.

### Optical Character Recognition

The original data files are PDF documents containing typed safety plans without handwritten content. We applied Tesseract [13], an open-source text recognition engine, to extract text for LLM development. After converting the PDF files to TIFF format using the Python Imaging Library (Python Software Foundation), we applied Tesseract version 5 with default configurations for English text extraction, generating plaintext output.

### Parsing

The original dataset included safety plan responses along with metadata (eg, step titles, operational definitions, and page numbers). First, to focus solely on responses, metadata elements were removed. Second, documents with clear “Step X” headings (eg, “Step 1. Warning signs”) were parsed directly to extract the response content for each step. In cases without explicit step markers, pattern matching based on recurring labels (eg, “Warning signs: 1., 2.… Internal Coping Strategies: 1., 2.…”) was used to segment the responses. Third, for the final step (eg, “Step 7: Reasons for living”), which lacks a clear ending marker (such as a “Step 8”), we used other textual indicators appearing after the last step (eg, “The Stanley-Brown Safety Plan...”) to identify the end of the response. Finally, after extracting the responses for each step, they were further processed because each response requires separate scoring. If the responses were numbered (eg, “1. 2. 3.”), this numbering was used to split and identify each response individually. In instances where the responses were not numbered, the text was divided by new line characters, and each line was treated as a distinct response.

### Prompt Development and Scoring Algorithm

We used a zero-shot prompting strategy to rate each response and compared the model performance of three scoring systems. The prompt was developed based on the SPISA coding manual and iteratively refined to enhance the model’s performance throughout the process. We tested 3 scoring systems: the original scoring system based on SPISA, a high-precision system, and a low-to-moderate precision system. The 4-score original system consists of four levels: (0) no response, (1) general, (2) somewhat specific, and (3) highly specific. Examples of responses with varying levels of precise specificity and corresponding scores are provided in Textbox 1. The 3-score high precision system has (0) no response, (1) general, and (2) somewhat and highly specific. The 3-score low-to-moderate precision system has (0) no response, (1) general and somewhat specific, and (2) highly specific responses. To improve the explainability of the rater [14], we also incorporated reasoning generation for each score, ensuring that clinicians receive interpretable feedback.

Textbox 1. **Prompt example for step 1: warning signs.**

Instruction Prompt = '''Given the following responses, evaluate the quality of written personal "Warning signs among patients with suicide risk. Warning signs are specific thoughts, feelings, physiological states, or behaviors that may indicate suicidal crisis. For each response, return a score based on the criteria and provide clear reasoning for the score.

Criteria for scoring:

0 - No response provided, responses stating "Nothing provided" or "Nothing listed," or responses that do not clearly indicate that the clinician at least tried to elicit a response (e.g., "Client was very guarded", "Client declined to answer").

1 - Not relevant to warning signs of suicide (e.g., "Call therapist"), vague thoughts (e.g., "Bad thoughts"), unexplained emotions (e.g., "Moody", "Angry", "Sad", "Frustrated", "Stressed"), or unclear situations (e.g., "Relationship problems").

2 - Somewhat personalized. The response includes some specific details of the thoughts (e.g., "Repeated thoughts", "Don't want to talk to anyone"), feelings (e.g., "Start to get anxious", "Not in a good mood"), and situations (e.g., "Not sleeping well", "I get headaches", "Having argument with husband") but lacks enough detail to fully assess suicide risk.

3 - Highly personalized. The responses include specific thoughts (e.g., "Any suicidal thoughts, plans, or intentions," "When I think I can't take it anymore," "Thoughts like I want to be left alone, leave me alone"), detailed feelings (e.g., "Feeling trapped and stuck," "Feeling an overwhelming emptiness," "The feeling of having no one to talk to"), or intense symptoms (e.g., "Clenched fists," "Hearing voices", "Excessive sleep disturbance").

Combination responses: If a response combines multiple elements where each element would independently score a 2 (e.g., "Start to get anxious and start crying"), assign a score of 3 instead of 2. If a response combines multiple elements where each element would independently score a 1, assign a score of 1.

Response: {response_text}

Output:

Score: [score]

Reasoning: [Clearly outline the specific indicators used for assessment and explain how they are scored. Provide concrete examples or threshold criteria to justify your decision, making the prediction results more explainable]'''

### Large Language Models

Using Microsoft Azure Databricks, we assessed the performance of the Safety Plan Fidelity Rater (SPFR) tool using the SPISA-coded data and three LLMs: GPT-4, LLaMA 3, and o3-mini. We originally selected only GPT-4 because it tends to perform clinical coding tasks well, but we wanted to understand whether an open-weight model, such as LLaMA 3, was comparable in performance. In addition, o3-mini’s reasoning ability outperformed this baseline model. For GPT-4 and LLaMA 3, we compared models using 3 temperature settings (0.1, 0.5, and 0.9). For o3-mini, we evaluated performance across three reasoning effort levels (low, medium, and high). Four steps of the safety plan were evaluated by comparing the weighted *F*_1_-score (a measure of how accurately a model makes predictions by balancing the impact of different types of prediction errors and ensuring that categories with more data have a greater influence on the final score, with scores ranging from 0 to 100). In this study, we define the best-performing model as the one with the highest weighted *F*_1_-score, indicating the most accurate predictive performance. We selected *F*_1_-score because it is a recommended metric especially for multiclass prediction with imbalanced data [15], providing a more reliable measure of model performance by balancing both precision and recall. We additionally conducted a McNemar test to examine whether the best-performing model is significantly better statistically than the other LLMs.

In Textbox 1, we provide an example prompt for Step 1: warning signs using the original 4-value SPISA scoring system and Table 1 for the associated predictions and reasoning for each of the 3 LLMs.

**Table 1.** Examples of prediction results with reasoning for Step 1: warning signs for each large language model.

Large language model	Prediction results
GPT-4	Response: ‘excessive sleeping, hearing voices’ Rater’s score: 3 Large language model prediction score: 3 Reasoning: The response includes highly personalized and intense symptoms that are specific and clearly indicative of a suicidal crisis. “Excessive sleeping” and “hearing voices” are detailed and severe indicators that highlight significant distress and potential danger, both of which are crucial for assessing suicide risk. These elements align with the criteria for a score of 3, which encompasses specific and highly personalized thoughts, feelings, or symptoms.
LLaMA 3	Response: ‘excessive sleeping, hearing voices’ Rater’s score: 3 Large language model prediction score: 3 Reasoning: The response includes two specific indicators that are highly relevant to warning signs of suicide.
o3-mini	Response: ‘excessive sleeping, hearing voices’ Rater’s score: 3 Large language model prediction score: 3 Reasoning: The response includes two specific and detailed indicators. “Excessive sleeping” is a concrete behavioral or physiological sign, and “hearing voices” is an intense symptom that falls under highly personalized indicators. Both elements are explicitly mentioned in the criteria as examples of intense or specific warning signs, making the overall response highly personalized. These details go beyond vague or general statements and clearly indicate significant risk factors, justifying a score of 3.

### Ethical Considerations

This study was approved by the University of Pennsylvania Institutional Review Board (852245) with the data use agreement and transfer (68141/02) from Columbia University.

## Results

### Data Characteristics

**Figure 2.**
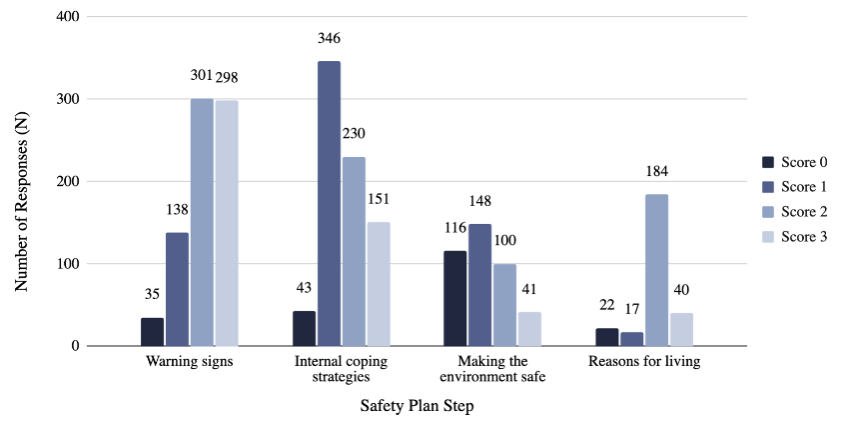
Distribution of responses by quality scores coded by trained raters.

The number of responses scored by coders, with scores of 0, 1, 2, or 3, displayed an asymmetrical distribution (Figure 2). For warning signs responses (n=772), the highest proportion of responses (301/772, 38.99%) received a score of 2, followed by 38.6% (298/772) scoring 3, 17.88% (138/772) scoring 1, and 4.53% (35/772) scoring 0. For internal coping strategies (n=770), 44.94% (346/770) of responses scored 1, followed by 29.87% (230/770) scoring 2, 19.61% (151/770) scoring 3, and 5.58% (43/770) scoring 0. Among responses on making the environment safe (n=405), 36.54% (148/405) scored 1, followed by 28.64% (116/405) scoring 0, 24.69% (100/405) scoring 2, and 10.12% (41/405) scoring 3. Finally, for reasons for living (n=263), most responses (69.96%, 184/263) scored 2, with 15.21% (40/263) scoring 3, 8.37% (22/263) scoring 0, and 6.46% (17/263) scoring 1 (Figure 2).

The prediction model for the quality scores of safety plan responses varied according to different scoring systems and LLMs (Table 2). We assessed four key questions: (1) How well do LLMs perform using the original scoring system? (2) Do LLMs have improved performance with high and low-to-moderate precision scoring systems? (3) Does one LLM outperform the others more consistently across steps? and (4) Which LLM is most consistent in its ratings?

**Table 2.** Weighted *F*_1_-score across large language models. Mean refers to the average weighted *F*_1_-score across different hyperparameters (eg, temperature for GPT-4 and LLaMA 3, reasoning effort for o3-mini).

SPI^a^ step		GPT-4					LLaMA 3					o3-mini				
		Mean	SD	Min	Max	Range	Mean	SD	Min	Max	Range	Mean	SD	Min	Max	Range
Step 1: warning signs																
Original		48.93	0.27	48.57	49.23	0.66	51.78	0.31	51.47	52.22	0.75	38.12	1.70	36.66	40.30	3.64
High		73.51	1.07	72.11	74.71	2.60	78.68	0.37	78.45	79.12^b^	0.67	59.53	0.79	59.03	60.46	1.43
L-t-M^c^		63.76	3.01	63.29	64.39	1.10	65.39	0.38	65.11	65.94	0.83	63.04	1.10	61.84	64.02	2.18
Step 2: internal coping strategies																
Original		53.27	0.59	52.70	54.08	1.38	60.19	0.54	59.59	60.65	1.06	58.86	1.84	56.56	60.73	4.17
High		64.22	0.54	63.52	64.85	1.33	72.77	0.55	72.11	73.18	1.07	70.50	1.63	68.67	72.57	3.90
L-t-M		76.92	0.77	76.08	77.94	1.86	79.33	0.17	79.13	79.53	0.40	80.15	1.65	78.30	81.13^b^	2.83
Step 6: making environment safe																
Original		56.84	0.38	56.32	57.24	0.92	58.52	0.14	58.35	58.68	0.33	59.73	0.87	58.52	60.52	2.00
High		64.33	0.33	63.92	64.72	0.80	67.38	0.19	67.16	67.61	0.45	67.90	0.72	66.86	68.55	1.69
L-t-M		75.01	1.17	73.61	75.73	2.12	74.40	0.56	73.74	74.82	1.08	74.38	1.96	72.96	76.56^b^	3.60
Step 7: reasons for living																
Original		75.79	1.68	73.65	77.22	3.57	78.86	0.58	78.38	79.52	1.14	74.47	4.55	69.26	78.81	9.55
High		88.23	2.07	85.67	89.97	4.30	90.69	0.27	90.38	91.02	0.64	91.72	0.24	91.42	91.99^b^	0.57
L-t-M		81.61	1.73	79.52	82.71	3.19	84.21	0.09	84.15	84.34	0.19	80.56	4.06	75.39	84.44	9.05

a SPI: safety planning intervention.

b The best-performing model for this step.

c L-t-M: low-to-moderate precision system scale.

How well do LLMs score the original scoring system? The mean performance (*F*_1_-score) for original scoring systems ranged by steps: warning signs: 38.12‐51.78; internal coping strategies: 53.27‐60.19; making environment safe: 56.84‐59.73; and reasons for living: 74.47‐78.86.

Do LLMs have improved performance with high and low-to-moderate precision scoring systems? We observed increases in predictive power with augmented precision scoring systems. The most elevated mean performance by scoring systems ranged by steps: warning signs (high): 59.53‐78.68; internal coping strategies (low-to-moderate): 76.92‐80.15; making environment safe (low-to-moderate): 74.38‐75.01; and reasons for living (high): 88.23‐91.72.

Does one LLM outperform the others more consistently across steps? LLaMA 3 produced the best predictive performance with the highest *F*_1_-score for warning signs (high): 79.12; o3-mini produced the best predictive performance for internal coping strategies (low-to-moderate): 81.13, making environment safe (low-to-moderate): 76.56, and reasons for living (high): 91.99.

Which LLM is most consistent in its ratings? LLaMA 3 (0.72) produced the most consistent performance, with the smallest mean range difference across different parameter values, compared to GPT-4 (1.99) and o3-mini (3.72).

### Best-Performing Model for Each Step

**Figure 3.**
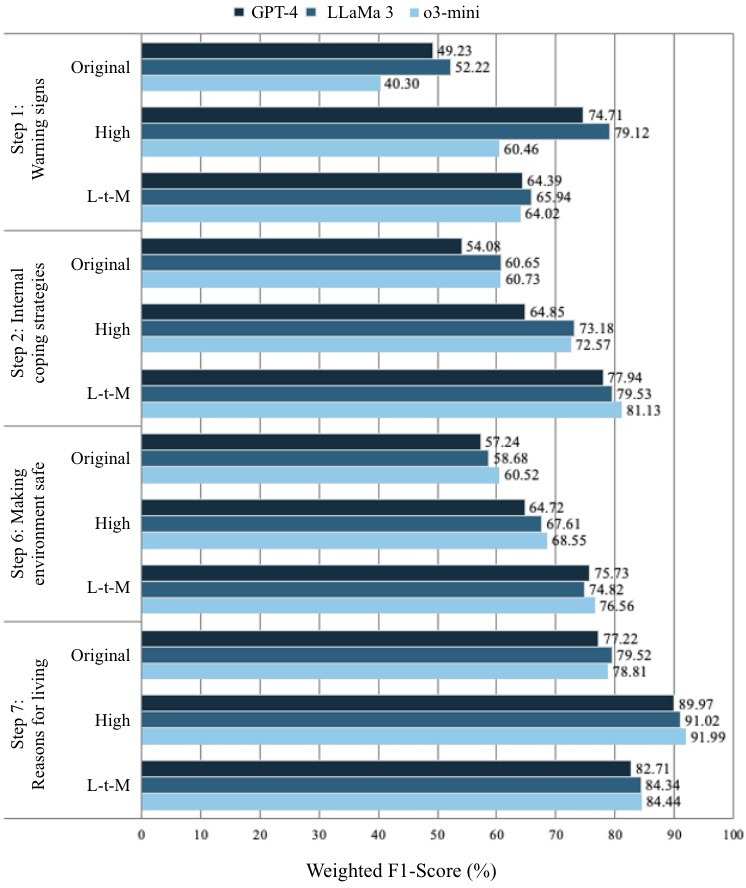
Best-performing model performance across steps, scoring systems, and large language models. L-t-M: low-to-moderate precision system scale.

The best-performing model for each step, defined as the model with the highest weighted *F*_1_-score across LLMs and scoring systems, was as follows (Figure 3). In addition, we conducted a McNemar test to examine whether the best-performing model, based on the highest weighted *F*_1_-score, differed significantly from the other LLMs. In other words, we compared the model with the highest weighted *F*_1_-score against each of the other models; for example, GPT-4 versus LLaMA 3, LLaMA 3 versus o3-mini, and o3-mini versus GPT-4.

- Step 1: Warning signs: The best-performing system was the high precision scoring system using LLaMA 3 with a temperature of 0.1, achieving a weighted *F*_1_-score of 79.12%. According to the McNemar test result, this LLaMA 3-based model showed a statistically significantly higher *F*_1_-score than both GPT-4 (*P*=.01) and o3-mini (*P*<.001).
- Step 2: Internal coping strategies: The best-performing system was the low-to-moderate precision scoring system using o3-mini with medium reasoning effort, achieving a weighted *F*_1_-score of 81.13%. This model showed a statistically significantly higher *F*_1_-score than GPT-4 (*P*<.001), but no significant difference compared to LLaMA 3 (*P*=.25).
- Step 6: Making the environment safe: The best-performing system was the low-to-moderate precision scoring system using o3-mini with medium reasoning effort, achieving a weighted *F*_1_-score of 76.56%. Although this model reported the highest weighted *F*_1_-score among the 3 LLMs, there were no statistically significant differences in *F*_1_-score compared to GPT-4 (*P*=.08) or LLaMA 3 (*P*=.39).
- Step 7: Reasons for living: The best-performing system was the high precision scoring system using o3-mini with medium reasoning effort, achieving a weighted *F*_1_-score of 91.99%. This model reported no statistically significant differences in *F*_1_-score compared to GPT-4 (0.87) and LLaMa 3 (*P*=.10)*.*

**Figure 4.**
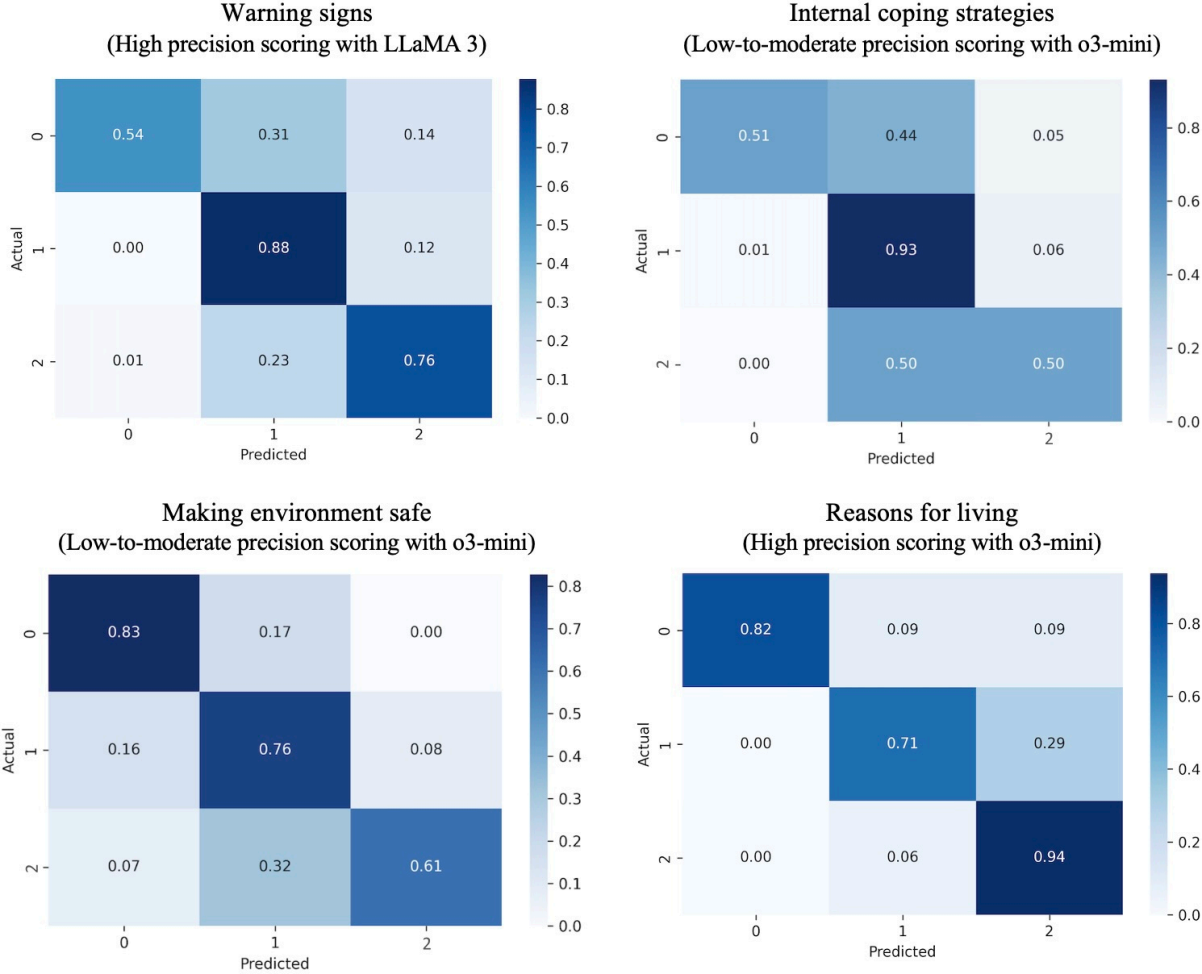
Confusion matrix of the best-performing large language model.

The confusion matrix in Figure 4 illustrates how well the top-performing models’ predicted scores align with the raters’ scores across different categories. For Step 1, warning signs responses with the high precision scoring system, among those scored as 0 by coders, 54% (19/35) were correctly predicted as 0 by the LLM model, while 31% (11/35) were misclassified as 1, and 14% (5/35) were misclassified as 2. Among responses scored as 1, 88% (121/138) were correctly predicted as 1, while 12% (17/138) were misclassified as 2. Among responses scored as 2, 76% (455/599) were correctly predicted as 2, while 1% (6/599) were misclassified as 0, and 23% (138/599) were misclassified as 1.

For Step 2, internal coping strategies responses with the low-to-moderate precision scoring system, among those scored as 0 by coders, 51% (22/43) were correctly predicted as 0 by the LLM model, while 44% (19/43) were misclassified as 1, and 5% (2/43) were misclassified as 2. Among responses scored as 1, 93% (536/576) were correctly predicted as 1, while 1% (6/576) were misclassified as 0, and 6% (35/576) were misclassified as 2. Among the responses rated as 2, 50% (76/151) were correctly predicted as 2, while 50% (76/151) were misclassified as 1.

For Step 6, making the environment safe responses with the low-to-moderate precision scoring system, among those scored as 0 by coders, 83% (96/116) were correctly predicted as 0 by the LLM model, while 17% (20/116) were misclassified as 1. Among responses scored as 1, 76% (188/248) were correctly predicted as 1, while 16% (40/248) were misclassified as 0, and 8% (20/248) were misclassified as 2. Among responses scored as 2, 61% (25/41) were correctly predicted as 2, 7% (3/41) were misclassified as 0, and 32% (13/41) were misclassified as 1.

For Step 7, reasons for living responses with the high precision scoring system, among those scored as 0 by coders, 82% (18/22) were correctly predicted as 0 by the LLM model, while 9% (2/22) were misclassified as 1, and another 9% (2/22) were misclassified as 2. Among responses scored as 1 by coders, 71% (12/17) were correctly predicted as 1, while 29% (5/17) were misclassified as 2. Among responses scored as 2, 94% (211/224) were correctly predicted as 2, and 6% (13/224) were misclassified as 1.

**Figure 5.**
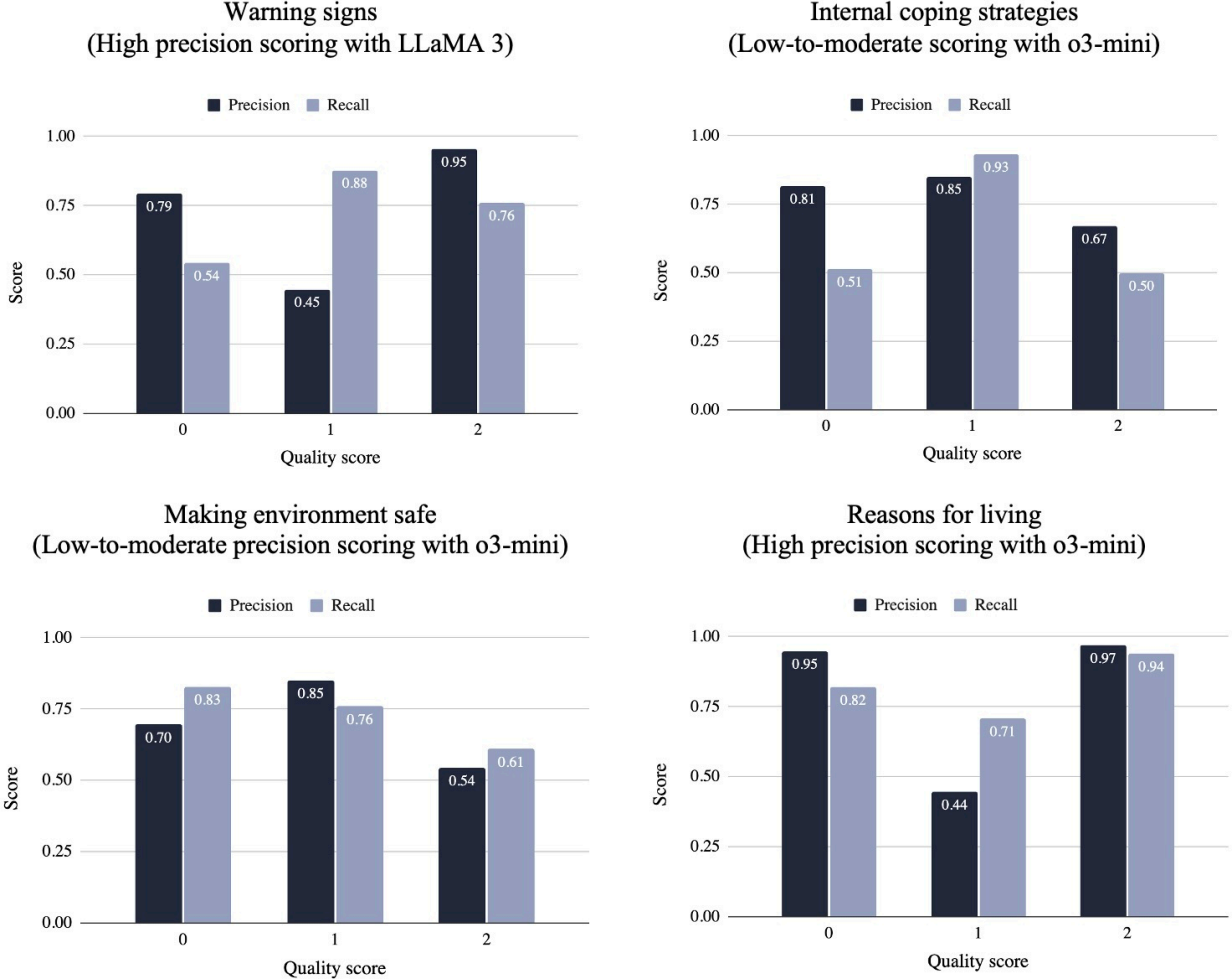
Precision and recall of the best-performing large language model.

The results across different steps indicate variations in precision and recall for each score value (Figure 5). In Step 1, warning signs, Score 0 demonstrated moderate precision (0.79) and low recall (0.54); Score 1 had low precision (0.45) yet high recall (0.88); and Score 2 had high precision (0.95) with moderate recall (0.76). In Step 2, internal coping strategies, Score 0 achieved high precision (0.81) but had low recall (0.51); Score 1 showed both high precision (0.85) and recall (0.93); and Score 2 showed both low precision (0.67) and recall (0.50). In Step 6, making the environment safe, Score 0 had a moderate precision (0.70) and high recall (0.83); Score 1 had high precision (0.85) and moderate recall (0.76); and Score 2 had both low precision (0.54) and recall (0.61). In Step 7, reason for living, Score 0 had both high precision (0.95) and recall (0.82); Score 1 had low precision (0.44) but moderate recall (0.71); Score 2 had both high precision (0.97) and recall (0.94).

## Discussion

### Principal Findings

We assessed the performance of 3 LLMs for scoring written safety plans. SPFR accuracy improved when using the 3-point scoring systems compared to the original 4-point scoring system. No one LLM provided the most optimal performance across steps and scoring systems. The findings of this study offer significant methodological advancements and areas for future research, particularly as they apply to the clinical implications of this line of research.

From a clinical practice perspective, while existing LLM applications in suicide prevention focus on screening, diagnosis, or delivering eHealth services to patients [9,16], to our knowledge, this study is innovative by demonstrating the utility of LLM in assessing written safety plan quality. At this stage, this work is too premature to apply different scoring systems practically, and further research is needed to determine the best LLM and scoring system to deploy across all steps of the safety plan. Future research will also evaluate the associations of the different scoring systems with patient outcomes (eg, suicidal behavior) and determine if changing the original scoring system is useful. A deeper understanding of different LLM-based scoring systems across all the steps of the safety plan and their clinical implications is essential for optimizing the provision of reliable, accurate feedback to clinicians. Specifically, given the preliminary nature of this work, further research is needed to optimize and select the best LLM model for scoring the entire written safety plan, as this pilot work only focused on four of the seven steps. In addition, before modifying the original scoring system to improve rating performance, research is needed to understand how potential modifications in scoring might impact associations with patient outcomes (eg, suicidal behavior) to determine if changes to the scoring system are both face valid and warranted. For instance, we can test the hypothesis that the SPFR implemented with the high precision scoring system predicts patient suicidal behavior with greater accuracy than the SPFR implemented with the low-to-moderate scoring systems. Beyond predictive accuracy and associations with patient outcomes, future research should also explore different implementation strategies for providing feedback to clinicians in order to design a tool that is most useful for clinical practice. The ultimate goal of this line of research is to establish that the automatic scoring system that is designed can enhance the quality of written safety plans. Incorporating qualitative evaluations, such as experts’ agreement with LLMs’ reasoning, can further improve the interpretability and acceptability of AI-generated feedback. Furthermore, embedding this tool within electronic health records systems would enable direct integration with documented safety plans, improving intervention quality by providing timely and actionable feedback to clinicians. For example, an integrated SPFR in the electronic health records that automatically rates safety plans and provides pop-up feedback to the clinician in real time may lead to increases in the quality of patient safety plans, which, in turn, may result in further reducing suicide risk for those patients who receive this intervention.

From a methodological perspective, this study is a first step in a line of inquiry about engineering questions to consider when designing an automatic scoring tool, such as the SPFR, using medical record data. At this phase, the reported LLM models remain under experimental testing, and future work is needed to evaluate and improve their clinical utility. Beyond selection of which LLMs to assess, how to set the scoring systems, and which are most consistent in their ratings, other potential considerations include: (1) selecting one LLM model for all 7 steps of safety plan or select the best-performing model for each step, (2) creating an LLM ensemble-based voting approach to assign a single score for each step, and (3) introducing few-shot learning and emerging methodologies to optimize LLM performance. Further, ethical considerations will be essential for the use of this tool in clinical practice. For instance, improving the transparency of LLM predictions can help clinicians understand the rationale behind the scores better, thereby supporting informed and responsible decision-making. In addition, evaluating these tools using data from more diverse and generalizable samples will be important for reducing bias and promoting the fairness of AI.

This study has several limitations. First, we assessed a selected sample, which consisted of typed safety plans that closely aligned with the Stanley-Brown Safety Plan form. Hence, these findings may not generalize to settings where safety plans are handwritten or vary in formatting. Our post hoc evaluation using the McNemar test revealed that performance differences between the LLMs (GPT-4, LLaMa 3, and o3-mini) were only partially significant. Therefore, it may be premature to draw definitive conclusions about which LLM performs best. Further evaluation with larger and more generalizable datasets is recommended.

### Related Works

This study is one of the few, but critical, emerging works in automated methods for scoring, characterizing, and assessing the efficacy of written safety plans. Boggs et al [11] developed a natural language processing and rules-based system based on the ConText algorithm for identifying documented professional contacts, lethal means counseling for firearms, and lethal means counseling for medication access and storage from safety plans. Our study builds upon this work as the first study to apply LLMs to automatically score the quality of safety plans.

### Conclusions

From this pilot project, we conclude that LLMs have the potential to support an automatic SPFR system and have identified clear paths toward improving LLM scoring performance and SPFR methodological development.

## Abbreviations

AI: artificial intelligence
LLM: large language model
SPFR: Safety Plan Fidelity Rater
SPI: safety planning intervention
SPISA: Safety Planning Intervention Scoring Algorithm
